# The Impact of *Bdnf* Gene Deficiency to the Memory Impairment and Brain Pathology of APPswe/PS1dE9 Mouse Model of Alzheimer’s Disease

**DOI:** 10.1371/journal.pone.0068722

**Published:** 2013-07-03

**Authors:** Tomi Rantamäki, Susanna Kemppainen, Henri Autio, Saara Stavén, Hennariikka Koivisto, Masami Kojima, Hanna Antila, Pasi O. Miettinen, Elisa Kärkkäinen, Nina Karpova, Liisa Vesa, Lothar Lindemann, Marius C. Hoener, Heikki Tanila, Eero Castrén

**Affiliations:** 1 Neuroscience Center, University of Helsinki, Helsinki, Finland; 2 A.I.Virtanen Institute, University of Eastern Finland, Kuopio, Finland; 3 Research Institute for Cell Engineering, National Institute of Advanced Industrial Science and Technology, Osaka, Japan; 4 Discovery Neuroscience, F. Hoffmann-La Roche Ltd., Basel, Switzerland; 5 Department of Neurology, Kuopio University Hospital, Kuopio, Finland; Cedars-Sinai Medical Center, Maxine-Dunitz Neurosurgical Institute, United States of America

## Abstract

Brain-derived neurotrophic factor (BDNF) importantly regulates learning and memory and supports the survival of injured neurons. Reduced BDNF levels have been detected in the brains of Alzheimer’s disease (AD) patients but the exact role of BDNF in the pathophysiology of the disorder remains obscure. We have recently shown that reduced signaling of BDNF receptor TrkB aggravates memory impairment in APPswe/PS1dE9 (*APdE9*) mice, a model of AD. The present study examined the influence of *Bdnf* gene deficiency (heterozygous knockout) on spatial learning, spontaneous exploratory activity and motor coordination/balance in middle-aged male and female *APdE9* mice. We also studied brain BDNF protein levels in *APdE9* mice in different ages showing progressive amyloid pathology. Both *APdE9* and *Bdnf* mutations impaired spatial learning in males and showed a similar trend in females. Importantly, the effect was additive, so that double mutant mice performed the worst. However, *APdE9* and *Bdnf* mutations influenced spontaneous locomotion in contrasting ways, such that locomotor hyperactivity observed in *APdE9* mice was normalized by *Bdnf* deficiency. Obesity associated with *Bdnf* deficiency did not account for the reduced hyperactivity in double mutant mice. *Bdnf* deficiency did not alter amyloid plaque formation in *APdE9* mice. Before plaque formation (3 months), BDNF protein levels where either reduced (female) or unaltered (male) in the *APdE9* mouse cortex. Unexpectedly, this was followed by an age-dependent increase in mature BDNF protein. *Bdnf* mRNA and phospho-TrkB levels remained unaltered in the cortical tissue samples of middle-aged *APdE9* mice. Immunohistological studies revealed increased BDNF immunoreactivity around amyloid plaques indicating that the plaques may sequester BDNF protein and prevent it from activating TrkB. If similar BDNF accumulation happens in human AD brains, it would suggest that functional BDNF levels in the AD brains are even lower than reported, which could partially contribute to learning and memory problems of AD patients.

## Introduction

The transgenic APPswe/PS1dE9 (*APdE9*) mouse line is a widely used model of Alzheimer’s disease (AD). Specific mutations in amyloid precursor protein (*APP*; K595N, K596L) and *presenilin-1* (exon 9 deletion) genes in these mice lead to altered proteolytic processing and metabolism of APP in brain which further lead to increased production of highly insoluble amyloid-β aggregates. Consequently, amyloid plaques gradually develop in the cortex and hippocampus of *APdE9* mice starting at 4 months of age [1], which is followed by memory impairment 4–8 months later [2], [3]. Thus, these mice recapitulate the order of pathological events in AD patients, in whom amyloid plaque formation can progress for years before memory impairment becomes manifested, as revealed by recent PET imaging studies with amyloid binding ligand [4], [5]. The *APdE9* mice therefore offer an excellent model for studying the molecular mechanisms downstream of the amyloid plaque formation and subsequent memory impairment.

Neurotrophins (NGF, nerve growth factor; BDNF, brain-derived neurotrophic factor; NT-3, neurotrophin-3; NT-4, neurotrophin-4) are a small family of secreted trophic proteins that critically regulate the survival and differentiation of specific subpopulations of neurons in the peripheral nervous system during early development [6]. In the adult brain NGF preferentially supports cholinergic neurons located in basal forebrain [7]–[9]. On the other hand, the role of BDNF in the regulation of synaptogenesis and neuronal plasticity and maintenance in several brain areas and neuronal subsystems has been well characterized [6], [10], [11].

Growing body of evidence suggests that neurotrophin mediated trophic support is reduced in AD [12]. For example, the levels of pro-apoptotic form of NGF, pro-NGF, are increased in AD patients [13]. Moreover, reduced levels of BDNF mRNA and protein (pro and mature) have been detected in AD patients and in some animal models of AD [14]–[23]. However, one study reported increased BDNF protein levels in AD brains [24].

Previous studies indicating abnormal neurotrophin signaling in AD together with the known ability of neurotrophins to support survival of their target neurons after injury has led to a suggestion that neurotrophins could prevent or delay neuronal and synaptic loss seen in neurodegenerative disorders, including AD [25]. Indeed, NGF delivery into the brain ameliorates memory impairment associated with old age [8] and amyloid-β infusion [26] in rats, and most importantly, clinical AD [27]. Furthermore, viral transduction or direct infusion of BDNF protein in the entorhinal cortex has been shown to reverse neuronal atrophy and synapse loss in an AD mouse model as well as in aged rats and primates [28]. In line with these observations we have recently shown that overexpression of the dominant-negative TrkB receptor, TrkB.T1, in *APdE9* mice exacerbated their spatial memory impairment at 12 months of age while the overexpression of catalytic TrkB receptors alleviated it [29]. The present study addressed the next logical question whether a decrease in the main TrkB ligand, BDNF, would also lead to aggravated spatial memory impairment in AD model mice, which would further indicate its role in AD pathology. To this end, we crossed *APdE9* mice with BDNF deficient mice (*Bdnf* ^+/−^), and tested spatial learning and memory of these mice in the Morris swim task at the age of 12 months. We also examined the brain BDNF signaling (protein, mRNA, p-TrkB) in *APdE9* mice during different stages of pathology.

## Materials and Methods

### Animals

The APPswe/PS1dE9 (*APdE9*) founder mice were obtained from Johns Hopkins University, Baltimore, MD, USA (D. Borchelt and J. Jankowsky, Dept. Pathology) and a colony was established at the University of Eastern Finland (Kuopio, Finland). These mice were generated by co-injection of chimeric mouse/human APPswe (mouse APP695 harboring a human Aβ domain and mutations K595N and M596L linked to Swedish familial AD pedigrees) and human PS1-dE9 (deletion of exon 9) vectors controlled by independent mouse prion protein promoter elements [30]. This mouse line was originally maintained in a hybrid C3HeJ × C57BL6/J F1 background, but the mice used in the present study were derived from backcrossing to C57BL6/J for 12 generations. The development of *Bdnf* ^+/−^ heterozygote knock-out mice (C57BL6/J background) have been described previously by Ernfors *et al* [31] (obtained from Jackson Laboratory). Crossing of these two mouse lines yielded four different genotypes, indicated as:
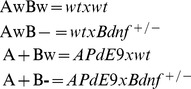



The housing conditions (National Animal Center, Kuopio, Finland) were controlled (temperature +22°C, light from 07∶00 to 19∶00; humidity 50–60%), and fresh food and water were available *ad libitum*. All behavioral tests were conducted between 9∶00–16∶00. The experiments were conducted according to the Council of Europe (Directive 86/609) and Finnish guidelines, and approved by the State Provincial Office of Eastern Finland (animal permit: ESAVI-10-05581). All experiments were designed and conducted in manner minimizing the use (e.g. sufficient number of animals per group) and suffering of animals.

The animals used in the behavioral testing started the tests at the age of 12 months and were sacrificed at the age of 13 months. The number of animals in each behavioral test and *port mortem* assay is given in **Table 1** and **Table 2**.

**Table 1:** The number of animals of the *APdE9* × *Bdnf^+/−^* crossings included in the behavioral tests and *post mortem* assays.

	Behavior	Cortex	HC	Cortex	Amyloid	Tau
		BDNF	BDNF	BDNF		
		ELISA	ELISA	WB		
**Female**						
AwBw	13	13	13	3		
AwB−	16	16	16	3		
A+Bw	18	18	16	4		
A+B−	16	16	16	4		
**Male**						
AwBw	16					
AwB−	16					
A+Bw	16				5	8
A+B−	17				6	8

Abbreviations for genotypes: AwBw = *wt x wt*; AwB- = *wt x Bdnf^+/−^*; A+Bw = *APdE9 x wt*; A+B- = *APdE9 x Bdnf^+/−^*. Other abbreviations: BDNF = brain-derived neurotrophic factor; ELISA = enzyme-linked immunosorbent assay; HC = hippocampus; WB = western blot.

**Table 2:** The number of *APdE9* (A+) and wild-type (Aw) animals in the aging study on BDNF and NGF protein and TrkB phosphorylation levels.

	Cortex		HC		Cortex		Cortex		Cortex		HC	
	BDNF		BDNF		NGF		BDNF		pTrkB		pTrkB	
	ELISA		ELISA		ELISA		PCR		WB		WB	
	Aw	A+	Aw	A+	Aw	A+	Aw	A+	Aw	A+	Aw	A+
**Female**												
3 mo	6	5										
7 mo	6	7										
12 mo	12	12	12	12	8	7	7	9	9–12	10–12	12	12
13 mo	7	6										
15 mo	14	13–14	14	14								
**Male**												
3 mo	6	6										
9 mo	8	9										
15 mo	6	6										

Abbreviations: BDNF = brain-derived neurotrophic factor; ELISA = enzyme-linked immunosorbent assay; HC = hippocampus; NGF = nerve growth factor; PCR = polymerase chain reaction; WB = western blot; pTrkB = phosphorylated TrkB.

### Behavioral Testing

#### Morris swim task (water maze)

The Morris swim navigation task was used to test spatial learning and memory. The apparatus consisted of a black plastic pool with a diameter of 120 cm and a black escape platform (14 cm×14 cm). The pool was filled with water 1.0 cm above the escape platform and the temperature of the water was constantly kept at 20±0.5°C. The mice were first pre-trained in two consecutive days to find and climb onto the submerged platform, aided by a guiding alley (1 m × 14 cm × 25 cm). In the testing phase (days 1–4), five 60-second trials per day were conducted with a recovery period of 2–5 min in a warmed cage between the trials. The location of the hidden platform was kept constant and the starting position varied between four different locations at the pool edge, with all mice starting from the same position in a given trial. Each mouse was placed in the water with its nose pointing towards the wall. If the mouse failed to find the escape platform within testing period, it was placed on the platform for 10 s by the experimenter (the same time was allowed for mice that found the platform). On day 5 the trial length was reduced to 40 s. In order to test the search bias the last trial on that day was run without the platform. A computer connected to an image analyzer (HVS Image®, Hampton, UK) calculated the escape latency to find the hidden platform, swim path length and the swimming speed. For the evaluation of thigmotaxis behavior the pool was divided into three concentric zones of equal surface area, and the time spent in the outer zone was calculated (the platform was located in the middle zone). The search bias during the probe trial was measured by calculating the time the mice spent in the vicinity of the former platform position, defined as target area (centered on the platform, diameter of 30 cm). This target area comprised 6.25% of the total surface area of the pool. Thus, if a mouse swims randomly in the pool it would expect to spend 2.5 s in the target area during the 40-second probe trial.

#### Spontaneous explorative activity

Spontaneous exploratory activity was analyzed in an observation cage (26×26×39 cm) with white opaque walls using infrared photo detection method coupled with an automated activity monitor (TruScan®, Coulbourn Instruments, CO, USA). The system was designed to enable separate monitoring of horizontal (XY-move time) and vertical activity (rearing). The test session took 10 min and was replicated after 48 h to assess the extent of habituation to the test cage. To avoid odor traces the test cage was cleaned with 70% ethanol before each mouse.

### Rotarod

The Rota-Rod® apparatus (Ugo Basile, Comerio, Italy) was used to test motor coordination and balance. The mouse was placed on a round rod (2 cm in diameter), the rotation of which gradually increased from 5 to 30 rpm. The latency to fall off (or turn two full rounds around with the rod) was recorded up till an 8-min cut-off time. The mouse was adapted to the test by first giving it 30 s to stay on a stationary rod and additional 30 s with the minimum speed of rotation.

### Biochemical Analysis

At the end of the experiment all female mice (see **Table 1**) were deeply anesthetized with pentobarbiturate-chloral hydrate cocktail (60 mg/kg each) and perfused transcardially with 50 ml heparinized ice-cold 0.9% saline (10 ml/min). The brain was removed and dissected on ice into the following blocks: frontal, parietal and temporal cortices, and hippocampus bilaterally. The tissue was snap frozen in liquid nitrogen and stored at –70°C. Frozen tissue samples were similarly collected in an additional age series from 3-, 7-, 9-, 13-, and 15-month-old *APdE9* and wild-type male or female mice (see **Table 2**).

#### Enzyme-linked immunosorbent assay (ELISA)

BDNF protein levels were assessed using ELISA as previously described [32], [33]. The assay preferentially detects mature BDNF over pro-BDNF and shows no cross-reactivity with other neurotrophins [33]. Briefly, brain samples were homogenized in NP++ buffer (137 mM NaCl, 20 mM Tris, 1% NP-40, 10% glycerol, 48 mM NaF, 2× Complete Inhibitor mix (Sigma-Aldrich, St. Louis, MO) and 2 mM Na_3_VO_4_), incubated on ice for 15 minutes, centrifuged (16000 g, 15 min, +4°C) and the supernatants were collected. Next the samples were diluted (1∶10-15) in Hanks buffer (125 mM NaCl, 5 mM KCl, 1.2 mM NaH_2_PO_4_, 1 mM CaCl_2_, 1.2 mM MgCl_2_, 1 µM ZnCl_2_, 10 mM Glucose, 25 mM HEPES, 0.25% BSA; pH 7.4), acidified (pH 2-3) with 1N HCl and after 15 minutes neutralized with 1N NaOH. BDNF standards, samples (all 170 µl) and POD-conjugated secondary mouse monoclonal BDNF antibody (mAb#9, see Ref. 32; 30 µl, 1∶1900 in Hanks containing 6.66% BSA and 0.66% Triton X-100) were transferred to pre-blocked (Hanks buffer, 2% BSA, 0.1% Triton X-100) Nunc Maxisorb ELISA (Thermo Fisher Scientific, Vantaa, Finland) plates that were previously coated with the primary mouse monoclonal BDNF antibody (mAb#1, see Ref. 32; 1∶4000 in Carbonate buffer, over night, +4°C). Next morning the plates were extensively washed with PBS-T and the colorimetric reaction was initiated, terminated and counted according to manufacturer′s instructions (BM Blue, Roche Diagnostics Oy, Espoo, Finland). BDNF content was calculated according to the standard curve. The r^2^ for the standard curve was ≥0.98 in all experiments. The BDNF ELISA assay was further validated by running separate analyses with hippocampal tissues obtained from conditional BDNF knock-out mice [34] and hippocampal tissues obtained from mice chronically treated with fluoxetine [35], an antidepressant drug that has been shown to slowly facilitate BDNF synthesis in hippocampus [36]. Sets of results were confirmed using a commercially available BDNF ELISA kit according to manufacturer′s instructions (Quantikine human BDNF ELISA kit, R&D systems, Minneapolis, MN, USA).

#### Western blotting

For BDNF western blotting an equal volume of protein was mixed with 2× Laemmli buffer, heated and run in SDS-PAGE (sodium dodecyl sulphate polyacrylamide gel electrophoresis), under reducing conditions and transferred to a PVDF (polyvinylidene fluoride) membrane (Hybond-P, GE Healthcare, Buckinghamshire, UK). The membrane was blocked with 3% non-fat dry milk and incubated with a polyclonal BDNF antibody raised against the mature BDNF protein (1∶500, Catalog number sc-546, Santa Cruz Biotechnology, CA, USA). This antibody readily recognizes both pro and mature forms of BDNF and has been extensively used for the detection of BDNF in western blotting. The specificity of this antibody to recognize BDNF in western blotting was confirmed by employing hippocampal tissue lysates from cleavage-resistant BDNF knock-in mice (M. Kojima, personal communication), conditional BDNF knock-out mice [34], [37] and mice chronically treated with fluoxetine [35], as controls. After washing, the specifically bound antibodies were detected using HRP-conjugated anti-rabbit antibody and electrochemiluminescence (ECL) -based detection. For normalization Tuj1 (-tubulin) immunodetection was performed after membrane stripping (1∶1000, Babco, Richmond, CA, USA). Band intensity was analyzed using ImageJ software (NIH, Bethesda, MD, USA).

For TrkB receptor tyrosine phosphorylation analysis an equal amount of protein was incubated with wheat germ agglutinin agarose (Catalog number A-2101-25, EY laboratories, San Mateo, CA, USA) for 1.5 hours at +4C. Next the beads were washed with NP++ buffer, specifically bound proteins eluted with 2× Laemmli buffer, separated in SDS-PAGE and transferred to PVDF membrane. After blocking the membrane was incubated with a polyclonal phospho-TrkB^Y816^ antibody raised in rabbits (1∶1000; a kind gift from Dr. Moses Chao, Skirball Institute, New York, USA). The generation and validation of this antibody has been characterized in previous publications [38]–[40]. After washing specifically bound antibodies were detected using HRP-conjugated anti-rabbit antibody and ECL -based detection. For normalization TrkB immunodetection (1∶1000, Catalog number 610101, BD Transduction Laboratories, Franklin Lakes, NJ, USA) was performed after membrane stripping. Band intensity was analyzed using ImageJ software.

#### Reverse transcription polymerase chain reaction (RT-PCR)

The level of total *Bdnf* mRNA was analyzed using RT-PCR as described in Karpova *et al* [41]. Briefly, after Trizol –based RNA extraction (Invitrogen, Carlsbad, CA, USA), total RNA was treated with DNAse I mix (Fermentas GmbH, Helsinki, Finland) and then reverse transcribed using oligo(dT) primer and SuperScript III Reverse Transcriptase mix (Invitrogen, Carlsbad, CA, USA). The control reactions without Reverse Transcriptase were also performed. The amount of cDNA was quantified using LightCycler SYBR-Green 1 Master mix (Roche Diagnostics Oy, Espoo, Finland) by real-time PCR. Total *Bdnf* cDNA was amplified using the following primers: 5′-GAAGGCTGCAGGGGCATAGACAAA-3′ and 5′-TACACAGGAAGTGTCTATCCTTATG-3′. For normalization, GAPDH cDNA levels were analyzed with the following primers 5′-GGTGAAGGTCGGTGTGAACGG-3′ and 5′-CATGTAGTTGAGGTCAATGAAGGG-3′. Ct values from each sample were obtained using the LightCycler 480 software (Roche Diagnostics Oy, Espoo, Finland).

### Histological Analysis

At the end of the experiment all male mice (see **Table 1**) were deeply anesthetized with pentobarbiturate-chloral hydrate cocktail (60 mg/kg each) and perfused transcardially with 50 ml heparinized ice-cold 0.9% saline (10 ml/min) followed by 4% paraformaldehyde. Brains were transferred to a 30% sucrose solution overnight and finally stored in a cryoprotectant in −20°C for later immunohistology. The brains were cut on a sliding/freezing microtome into 35 µm coronal sections. All sections were pretreated with sodium citrate solution at 80°C for 30 min.

To visualize the location of the strongest BDNF immunopositivity two sections at the level of mid-hippocampus were stained with the same specific BDNF antibody that was used as primary capturing antibody for ELISA assays (1∶1000, mAb#1). This antibody has been raised against mature BDNF and therefore cannot differentiate mature and pro forms of BDNF [32]. Importantly, this antibody does not recognize other members of neurotrophin family [32]. The sections were pretreated with 0.3% H_2_O_2_ in TBS-T for 30 min and then blocked in 1.5% normal goat serum in TBS-T for 1 h. The sections were incubated with the primary antibody overnight at room temperature on a shaker table. Following incubation, the sections were rinsed thoroughly with TBS-T and transferred to the solution containing the secondary antibody, biotinylated goat–anti mouse 1∶1500 (Catalog number BA-9200, Vector Laboratories, Peterborough, UK). After 2 hours of incubation the sections were rinsed three times and transferred to a solution containing Streptavidin 1∶1000 (GE Healthcare, Buckinghamshire, UK) for 2 hours. Visualization of BDNF-immunoreactivity was achieved by incubation with DAB–Ni solution. Stained sections were mounted on gelatin-coated slides and dehydrated in alcohol series, cleared with xylene and mounted in Depex.

To visualize amyloid plaque every 6th section in 5 A+Bw and 6 A+B- mice was stained with monoclonal mouse anti-human antibody W0-2 (Aβ4–10, 1∶30000, Genetics, Schlieren, Switzerland). The sections were incubated overnight at room temperature on a shaker table. Following incubation, the sections were rinsed thoroughly with TBS-T and transferred to a solution containing the secondary antibody, biotinylated goat–anti mouse (1∶1500; Vector Laboratories, Peterborough, UK). After 2 hours of incubation the sections were rinsed three times and transferred to a solution containing Streptavidin (1∶1000; GE Healthcare, Buckinghamshire, UK) for another 2 hours. Visualization of Aβ plaques was achieved by incubation with DAB–Ni solution. Stained sections were mounted on gelatin-coated slides and dehydrated in alcohol series, cleared with xylene and mounted in Depex.

To visualize dystrophic neurites around amyloid plaques with hyperphosphorylated tau we stained three frontal sections in 8 A+Bw and 8 A+B- mice with monoclonal anti-human PHF-Tau (AT8; 1∶1000, Thermo Scientific, Rockford, IL, USA). After citrate solution pretreatment, sections were blocked in 3% bovine serum albumin in TBS-T for 60 min. The primary antibody was also diluted into 3% BSA in TBS-T. The sections were incubated overnight at 4°C on a shaker table. Following incubation, the sections were rinsed thoroughly with TBS-T and transferred to the solution containing the secondary antibody, biotinylated goat–anti mouse (1∶1500; Vector Laboratories, Peterborough, UK). After 2 hours of incubation the sections were rinsed three times and transferred to a solution containing Streptavidin (1∶1000; GE Healthcare, Buckinghamshire, UK) for 2 hours. Visualization of antibody positivity was achieved by incubating the sections for 2 hours at room temperature in a solution containing CY3 red fluorescent dye (1∶1000; TSA- Plus Cyanine 3 System kit, PerkinElmer, Waltham, MA, USA). Stained sections were mounted on gelatin-coated slides and mounted in Vectashield (Vector Laboratories, Peterborough, UK).

For quantification of amyloid plaque load, three sections with 210 µm intervals were selected from the septal (rostral) half of the hippocampus. The sections were photographed using the Olympus BX40 microscope (Tokyo, Japan) with DP50 camera attached and images were treated and analyzed using Photoshop CS3 program (Adobe Systems Inc., San Jose, CA, USA). The images were transformed to grayscale and their brightness and contrast were changed using the shadow-highlight command (1 time for TK, maintaining the same threshold for all subgroups in a series). The area of the hippocampus was measured using the lasso-tool and hippocampal amyloid plaques were measured using the color range command (using threshold 110). The final value was obtained by dividing the amyloid plaque area by the total hippocampal area.

Because AT8-positive terminals were restricted to the immediate vicinity of amyloid plaques, we calculated their surface area relative to the plaque size. First, we identified the most posterior frontal section where corpus callosum splits (∼bregma +1.2) and selected those 15 amyloid plaques in the anterior cingulate cortex in each hemisphere that were closest to the midline. Based on the characteristic shape in the background (green) fluorescence we could identify the plaques and calculate their combined surface area. The total AT8-positive surface area (red fluorescence) was then calculated on the focal plane that gave the highest total area for each plaque. The total AT8-positive area was divided by the total plaque area and expressed as %.

As a control for nonspecific binding, amyloid plaques were also stained for anti-NGF, anti-CDNF antibodies, as well as for anti-GFAP to visualize activated astrocytes. We stained the section overnight using polyclonal rabbit anti-NGF (Santa Cruz, Dallas, TX, USA, cat.no SC-549) at 1∶2000 as the primary antibody. Goat anti-rabbit IgG biotinylated (Vector Laboratories, Peterborough, UK, cat.no BA-1000) at 1∶500 was used as the secondary antibody and streptavidin (GE Healthcare, Buckinghamshire, UK) at 1∶1000 as the tertiary antibody, both incubated for 2 h in TBS-T. Rabbit polyclonal antibody [42] was used for CDNF at 1∶1000 with an overnight incubation. The secondary and tertiary antibodies were the same as for NGF staining. Activated astrocytes were visualized by monoclonal anti-GFAP (clone G-A-5, Sigma-Aldrich, St. Louis, MO, USA, cat.no. G3893) at 1∶1000 with overnight incubation. Goat anti-mouse HRP (Pierce, Rockford, IL, USA, cat.no. 31430) at 1∶500 was used as the secondary antibody with a 2-h incubation. For all stainings, the pretreatment was as above: first 0.3% H_2_O_2_ in TBS-T for 30 min, then blocking in 1.5% normal goat serum in TBS-T for 1 h and visualization with DAB-NI solution.

### Statistical Analysis

The statistical analysis of behavioral tests was done using SPSS for Windows 14.0 software. The normal distribution of values for selected parameters in separate groups was tested using Kolmogorov-Smirnov Z one-sample test. The main means of analysis was two-way ANOVA with *APdE9* (A+ vs. Aw) and *Bdnf* ^+/−^ (B- vs. Bw) as between subject factors. When several time points were included as a within-subject factor, ANOVA for repeated measures was used with the same A and B factors. Males and females were always analyzed separately unless otherwise stated. However, in cases of borderline significance, we also assessed the effects with males and females combined. To assess the influence of body weight on sensible the behavioral test parameters, we ran the ANOVAs also with the body weight as a covariant. *Post hoc* analyses were done using Dunnett’s test with the AwBw groups as the reference. In cases where we focused on the effect of *Bdnf* deficiency alone, *Bdnf* ^+/−^ mice (B-) were compared to Bw mice of the corresponding *APdE9* gene status using Student’s t-test. The statistical analysis of biochemical tests were performed with Two-way Student’s t-test (comparison of two groups) or with One-way or Two-way ANOVA followed by Tukey-Kramer *post hoc* test. Statistical significance was set at p<0.05.

## Results

### *Bdnf* Gene Deficiency Leads to Increased Body Weight

As described in earlier studies [43], we found significantly increased body weight of *Bdnf* ^+/−^ mice when compared to wild-type mice in both genders (males: F_1,61_ = 15.7, p<0.001; females: F_1,58_ = 13.5, p = 0.001). The body weights at the end of the study among the males were as follows (in grams; mean±SEM): AwBw 34.5±0.9, AwB− 38.8±1.6, A+Bw 34.1±0.8, A+B− 39.2±1.2, and among the females follows: AwBw 27.2±0.6, AwB− 30.3±1.0, A+Bw 26.3±0.5, A+B− 29.1±0.9. The group sizes and labels are explained in **Table 1**. To take into account the possible confounding effect of body weight, we systematically replicated the statistics of the behavioral tests with the body weight as a covariant.

### Impact of *Bdnf* Gene Deficiency on Spatial Learning and Memory in Aged *APdE9* Mice

We crossed *APdE9* mice with *Bdnf* ^+/−^ mice and tested the spatial learning and memory of each resulting genotype in the Morris swim task at the age of 12 months. This age was selected based on our recent finding demonstrating that the over-expression of dominant-negative truncated TrkB receptor aggravates memory impairment in 12-month-old *APdE9* mice [29].

**Figure 1:**
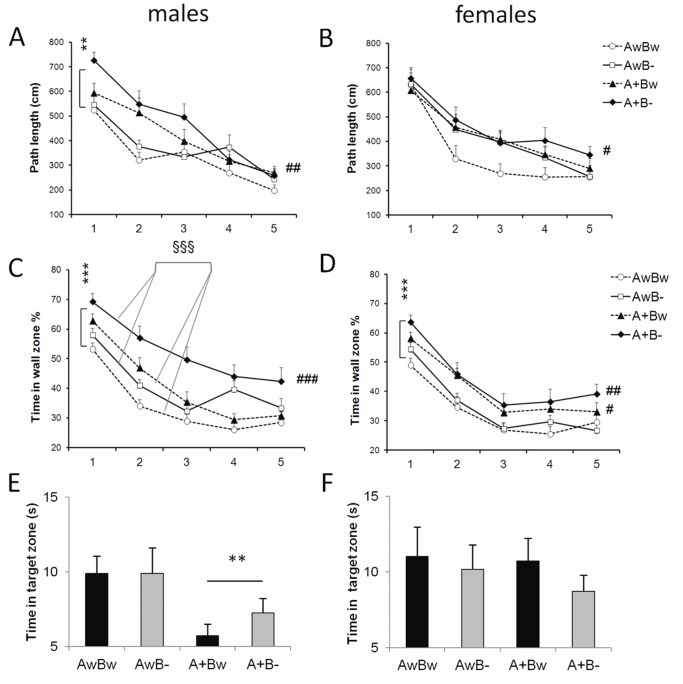
*Bdnf* gene deficiency aggravates memory impairment of *APdE9* mice in the Morris swim task. (**A**) Swim path length to the hidden platform in males; ** significant *APdE9* gene main effect (p = 0.002, ANOVA-rm), ^##^ A+B− mice differ significantly from the AwBw control group (p = 0.001, Dunnett’s post-hoc test). (**B**) Same in females; ^#^ A+B- mice differ significantly from the AwBw control group (p = 0.02, Dunnett’s post-hoc test). (**C**) Thigmotaxis calculated as percent time spent in the wall zone in males; *** significant *APdE9* gene main effect (p = 0.0002, ANOVA-rm), ^§§§^ *Bdnf* gene main effect (p = 0.0003, ANOVA-rm), ^###^ A+B− mice differ significantly from the AwBw control group (p<0.001, Dunnett’s post-hoc test). (**D**) Same in females; *** significant *APdE9* gene main effect (p = 0.0001, ANOVA-rm), ^##^ A+B− mice (p = 0.001) and ^#^ A+Bw mice (p = 0.03) differ significantly from the AwBw control group (Dunnett’s post-hoc test). The dashed line indicates a random performance. (**E**) Search bias (time in the target area in seconds) in the probe test without the platform on day 5 in males; ** significant *APdE9* gene effect (p = 0.007, ANOVA). (**F**) Same in females**.** Abbreviations for genotypes: AwBw = *wt x wt*; AwB− = *wt x Bdnf^+/−^*; A+Bw = *APdE9 x wt*; A+B- = *APdE9 x Bdnf^+/−^*.

As shown by numerous studies on aged *APdE9* transgenic mice before, both *APdE9* transgenic males (F_1,61_ = 17.2, p = 0.0001) and females (F_1,59_ = 4.7, p = 0.03) had longer escape latencies to the hidden platform when compared to corresponding wild-type mice. Importantly, *Bdnf* gene deficiency further strongly impaired learning in males (p = 0.007) and showed a similar trend in females (p = 0.07). However, among males, but not females, *Bdnf* gene deficiency on its own also reduced swimming speed (p = 0.02) that could partially contribute to significantly increased escape latency in males in this task. Reduced swimming speed in turn likely stemmed from obesity that rendered the mice more tolerant to cool water and more prone to float. Indeed, when body weight was included in the ANOVA model as a covariant, the impact of *Bdnf* gene deficiency on swimming speed in male mice also disappeared (p = 0.16). However, even when the influence of body weight was taken into account in the statistical model, male *Bdnf* ^+/−^ mice displayed longer escape latencies than those carrying both *Bdnf* alleles (p = 0.01). Furthermore, the *Bdnf* effect could be observed in both *APdE9* transgenic and wild-type mice (no interaction between the genotypes, all p values >0.51). To further analyze task acquisition with little confounding effect of swimming speed, we assessed the swim path lengths of mice (**Fig. 1** **A-B**). Similarly to escape latency, swim path length revealed impaired learning among *APdE9* mice (males: F_1,61_ = 12.9, p = 0.001; females: F_1,59_ = 4.7, p = 0.03). Both male and female *Bdnf* ^+/−^ showed a trend toward increased path length (p = 0.07, for both genders). However, when both sexes were combined, the effect of *Bdnf* gene deficiency became clearly significant (F_1,124_ = 7.2, p = 0.008; **Fig. S1A**). In addition, when the body weight was included in the ANOVA model, the effect of *Bdnf* gene deficiency was significant in both males (p = 0.01) and females (p = 0.04). Furthermore, in the *post hoc* tests, among for sexes only the double mutant *APdE9* × *Bdnf* ^+/−^ mice differed significantly from the double wild-type controls (**Fig. 1** **A–B**). There was no interaction between the genotypes in the swim path length in either sex or in the pooled analysis (all p-values >0.57).

Successful performance in the Morris water maze requires that the animals abandon their natural but fruitless effort to search for an escape in the pool wall. This thigmotaxic behavior was significantly influenced by the *APdE9* mutations in males (F_1,61_ = 15.7, p = 0.0002; **Fig. 1C**) and females (p = 0.0001, F_1,59_ = 16.8; **Fig. 1D**). *Bdnf* ^+/−^ males also displayed strong thigmotaxis (p = 0.0003; **Fig. 1C**) compared to mice with both *Bdnf* alleles, but this tendency was non-significant among females (p = 0.19; **Fig. 1D**). When both sexes were combined, the *Bdnf* main effect was significant (F_1,124_ = 13.8, p<0.001; **Fig. S1B**). Notably, among both males and females, mice carrying both *APdE9* and *Bdnf* mutations seemed to have the strongest thigmotaxis (**Fig. 1C–D**).

Memory of the platform location was tested on the last day with a probe trial without the platform and assaying the search bias of the mice. Among males, *APdE9* mutant mice showed clearly impaired search near the former platform location (p = 0.007; **Fig. 1E**), while such genotype difference was not present among females (p = 0.58; **Fig. 1F**). In contrast to task acquisition, memory for the platform location was not dependent on the *Bdnf* gene status (males: p = 0.53; females: p = 0.36).

### *Bdnf* Gene Deficiency Normalizes the Hyperactivity of *APdE9* Mice in a Novel Environment

**Figure 2:**
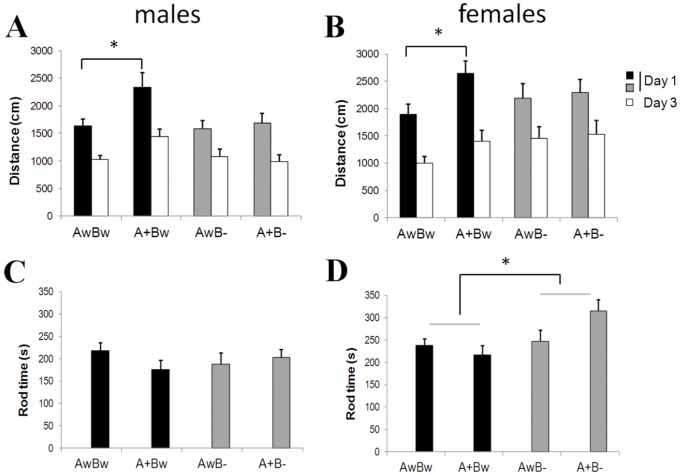
*Bdnf* gene deficiency normalizes the hyperactivity/hyperexploration of *APdE9* mice. Distance traversed (cm) in a novel test cage during 10 min on day 1 and day 3 (habituation) in males (**A**) and in females (**B**). * Significant *APdE9* gene effect among *Bdnf* wild-type mice, (p<0.05, ANOVA-rm). *Bdnf* gene deficiency does not impair motor coordination and balance despite obesity. Time on the accelerating rotarod in males (**C**) and in females (**D**). * Significant main effect of the *Bdnf* gene deficiency (p = 0.02, ANOVA). Abbreviations for genotypes: AwBw = *wt x wt*; AwB− = *wt x Bdnf^+/−^*; A+Bw = *APdE9 x wt*; A+B− = *APdE9 x Bdnf^+/−^*.

Our recent study showed that genetic inhibition of the TrkB receptor counteracts the hyperactivity present in *APdE9* mice [29]. Therefore, we assessed spontaneous exploration activity in a novel environment using a similar protocol and the same test cage as in our previous study [29]. As illustrated in **Fig. 2**, *APdE9* mutant mice traversed a longer distance during the 10-min test than wild-type mice. This was true for males (F_1,30_ = 7.4, p = 0.01; **Fig. 2A**) and females (F_1,28_ = 5.2, p = 0.03; **Fig. 2B**). This hyperactivity was lost in the double mutant *APdE9* × *Bdnf* ^+/−^ mice, both in males (F_1,31_ = 0.0, p = 0.99; **Fig. 2A**) and in females (F_1,30_ = 0.08, p = 0.78; **Fig. 2B**). The behavior of *Bdnf* ^+/−^ mice without *APdE9* mutations resembled the behavior of wild-type mice. The findings were only slightly changed when taking the body weight into account in the ANOVA model. The *APdE9* effect remained significant in the *Bdnf* wild-types males (p = 0.008) and was of borderline significance in females (p = 0.05). Furthermore, the *APdE9* effect was lost in the double mutant *APdE9* x *Bdnf* ^+/−^ mice (males, p = 0.95; females, p = 0.94). When the mice were subjected to the test two days later no differences were observed between the groups, thus demonstrating equal habituation to the testing environment in all genotypes.

### *Bdnf* Gene Deficiency Improves Motor Coordination in *APdE9* Mice

To further assess possible genotype differences in motor coordination and balance, we tested the ability of mice to stay on an accelerating rotating rod (Rotarod). Among male mice, *Bdnf* deficiently did not affect time on the rod (**Fig. 2C**), whereas among females there was a significant main effect of *Bdnf* (F_1,58_ = 5.9, p = 0.02) as well as a marginal *APdE9* x *Bdnf* interaction (p = 0.05). However, the difference was such that female *APdE9* x *Bdnf* ^+/−^ double mutant mice surprisingly outperformed the other groups in this task (**Fig. 2D**). In addition, when the body weight was taken into account in the ANOVA model, a significant *Bdnf* effect emerged in both sexes (both, p = 0.04). The *APdE9* x *Bdnf* interaction remained marginally significant in females (p = 0.05), but did not reach significance in males (p = 0.08). These observations clearly indicate that loss of hyperactivity in *APdE9 x Bdnf* ^+/−^ mice does not simply reflect difficulty to move around.

### *Bdnf* Gene Deficiency does not Alter Amyloid Pathology in *APdE9* Mice

**Figure 3:**
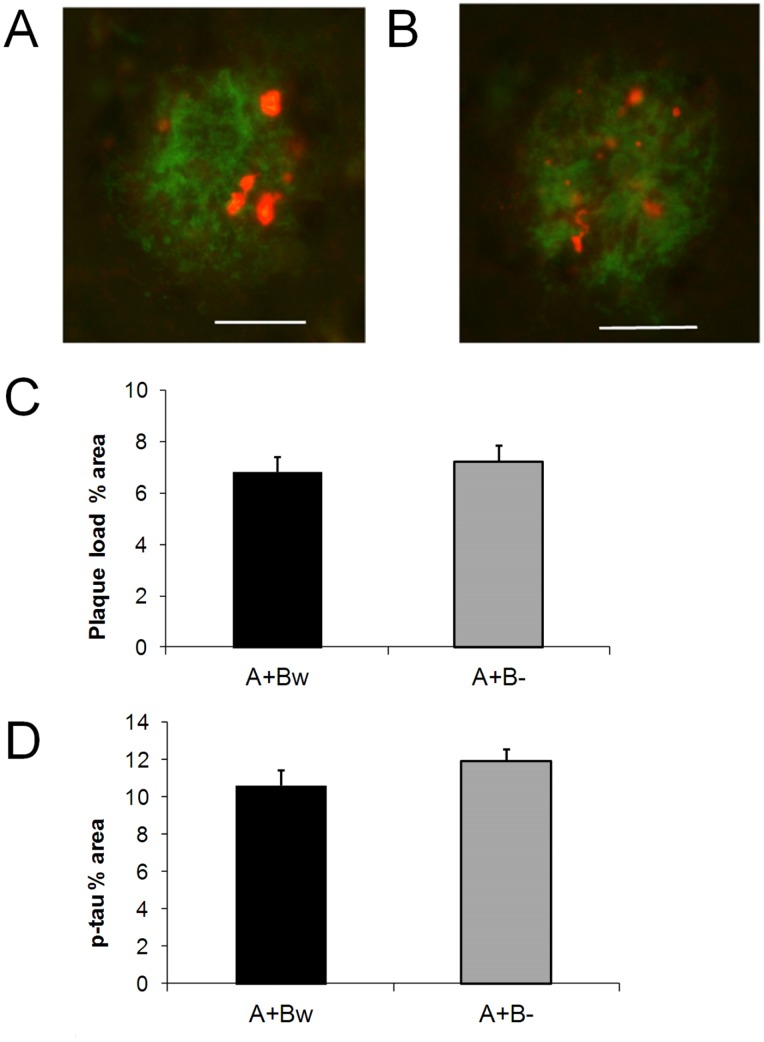
*Bdnf* gene deficiency does not alter amyloid pathology in *APdE9* mice. **A)** Example of an amyloid plaque (green) in the hippocampus surrounded by phosho-tau positive dystrophic neurites (red) in an *APdE9* mouse carrying both *Bdnf* alleles (A+Bw). Scale bar = 20 µm. **B)** Same in a mouse with only one *Bdnf* allele (A+B−). **C)** Amyloid load in the hippocampus expressed as % of examined surface area in *APdE9* mice carrying two *Bdnf* alleles (A+Bw) or only one (A+B−) allele. **D)** Phospho-Tau positive surface area around amyloid plaques in the anterior cingulate cortex (expresses as % of the combined plaque surface area) of *APdE9* mice carrying two (A+Bw) or one (A+B−) *Bdnf* alleles.

Next we analyzed the effect of *Bdnf* deficiency on amyloid pathology in *APdE9* mice. This analysis was done in male mice only. There was no difference between *Bdnf* ^+/−^ and *Bdnf* ^+/+^ carriers in the hippocampal amyloid load (t_9_ = 0.49, p = 0.64; **Fig. 3C**). Amyloid plaques in *APdE9* mice were surrounded by dystrophic neurites that stained positively for hyperphosphorylated tau (as revealed by AT8 antibody; **Fig. 3** **A, B**). The AT8-positive area around cortical plaques in the anterior cingulate cortex was not significantly different between *APdE9* x *Bdnf* ^+/−^ and *APdE9* x *Bdnf* ^+/+^ mice (t_14_ = 1.3, p = 0.22; **Fig. 3D**).

### Mature BDNF Protein Levels are Increased in Aged *APdE9* Mouse Brain

**Figure 4:**
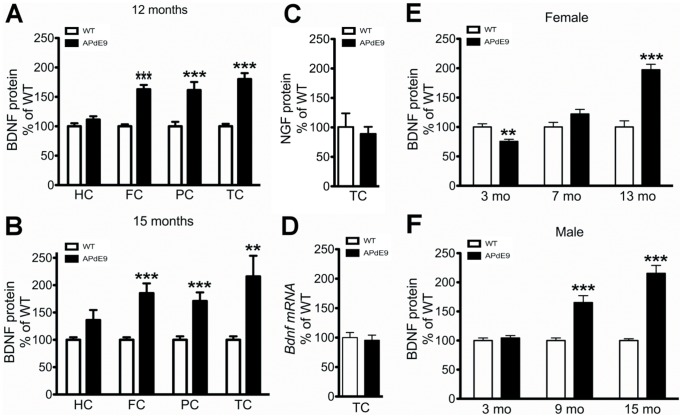
Age-dependent increase of BDNF protein in the brains of *APdE9* mice. **A)** BDNF protein levels (measured with ELISA) in the hippocampus, frontal cortex, parietal cortex and temporal cortex of 12-month-old female *APdE9* mice. **B)** BDNF protein levels (measured with ELISA) in the hippocampus, frontal cortex, parietal cortex and temporal cortex of 15-month-old female *APdE9* mice. **C)** NGF protein levels (measured with ELISA) in the temporal cortex of 12-month-old female *APdE9* mice. **D)** Total *Bdnf* mRNA (measured with RT-PCR) in temporal cortex of 12-month-old female *APdE9* mice. **E)** Age-dependent BDNF protein levels (measured with ELISA) in the temporal cortex of wild-type and *APdE9* female mice. **F)** Age-dependent BDNF protein levels (measured with ELISA) in the temporal cortex of wild-type and *APdE9* male mice. A t-test was performed between wild-type and mutant animals; *p<0.05, ** p<0.01, *** p<0.001.

Both reduced and increased *bdnf* mRNA and BDNF protein levels have been reported in different animal models of AD [23], [44], [45]. Thus, we sought to examine the regulation of BDNF signaling in *APdE9* mice during different stages of pathology. To this end we analyzed brain tissue of *APdE9* mice and wild-type littermates that were not part of the behavioral study. The ages, gender and number of animals are summarized in **Table 2**. We first determined brain BDNF protein levels using an ELISA assay in female *APdE9* mice at the age of 12 months, when memory impairment begins to manifest in these mice [3]. In agreement with some previous findings [45], BDNF protein levels were not reduced but slightly increased in the hippocampus of female *APdE9* mice (**Fig. 4A**). Interestingly, even more pronounced BDNF protein increases were observed in several cortical areas (**Fig. 4A**). Indeed, the increases in BDNF protein level were significantly higher in frontal cortex, parietal cortex and temporal cortex compared to hippocampus (all p values <0.005). Brain BDNF levels in female *APdE9* mice continued to increase toward 15 months age to the same extent in all brain areas studied (**Fig. 4B**), reaching over two-fold levels compared to WT mice in the temporal cortex. This increase in cortical samples seemed to be selective for BDNF, since temporal cortex samples of aged *APdE9* mice revealed no significant changes in NGF protein levels compared to WT littermates (**Fig. 4C**). Unexpectedly, the levels of total *Bdnf* mRNA in the cortical samples were not significantly different between middle-aged WT and *APdE9* mice (**Fig. 4D**).

To investigate the relationship between the observed increase in BDNF levels and amyloid accumulation in the *APdE9* mice, we examined BDNF protein levels with ELISA in temporal cortical samples of *APdE9* mice before the appearance of plaques. At the age of 3 months female *APdE9* mice showed a statistically significant reduction in BDNF levels and no significant differences were seen in males (**Fig. 4E–F**). At the age of 7 months, BDNF protein levels were not different from the wild-types in female *APdE9* mice, while at the age of 9 months, BDNF protein levels were significantly increased in male *APdE9* mice (**Fig. 4E–F**). This was followed by gradual and age-dependent increase in BDNF protein levels in the temporal cortex of *APdE9* mice in both sexes.

**Figure 5:**
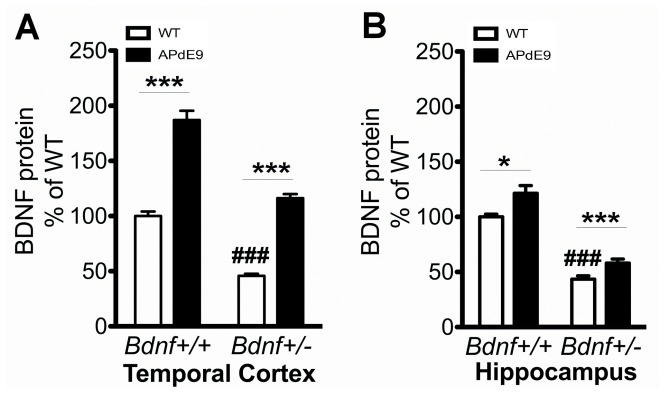
Impact of *Bdnf* gene deficiency on BDNF induction in 13-month-old female *APdE9* mouse cortex. **A)** **** BDNF protein levels in the temporal cortex of wild-type mice and mice carrying *APdE9* and/or *Bdnf* ^+/−^ mutations. **B)** BDNF protein levels in the hippocampus of wild-type mice and mice carrying *APdE9* and/or *Bdnf* ^+/−^ mutations. Two-Way ANOVA followed with Tukey-Kramer *post hoc* test was performed for statistical analysis. *p<0.05, ***p<0.001 compared to the respective wt, ###p<0.001 compared to wt/wt.

Next we assessed BDNF protein levels in female 13-month-old double mutant (*APdE9* x *Bdnf* ^+/−^) mice that have been previously subjected to behavioral tests. As expected, *Bdnf* ^+/−^ mice showed approximately 50% down-regulation of BDNF protein in the temporal cortex and hippocampus (**Fig. 5A–B**). Again, as revealed by ELISA, BDNF protein levels in *APdE9* mice were increased substantially in the temporal cortex and moderately in the hippocampus. Similarly, the levels of BDNF protein in the hippocampus and temporal cortex were increased to the same extent also in *APdE9* x *Bdnf* ^+/−^ mice compared to wt x *Bdnf* ^+/−^ mice, reaching the wild-type level in the temporal cortex (**Fig. 5A–B**). **Table 3** summarizes the correlations between individual brain BDNF levels and the key behavioral testing parameters. The only significant correlation was between BDNF levels in the temporal cortex and thigmotaxis, such that high BDNF levels were associated with strong tendency to keep swimming near the pool wall.

**Table 3:** Correlation between hippocampal (HC) and cortical (Ctx) BDNF protein levels and key behavioral test parameters in 13-month-old female mice.

	Ambulatory distance	Rod time	Mean esc. latency	Mean path length	Mean wall zone time	Search bias
HC BDNF	0.14	−0.18	0.21	0.14	0.18	0.21
Ctx BDNF	0.25	−0.12	0.29	0.16	**0.45** *	0.02

All four genotypes are pooled, with n = 29-31 in all correlations. Values are Spearman rho correlation coefficients.

* p = 0.01, p>0.10 for all other correlations.

**Figure 6:**
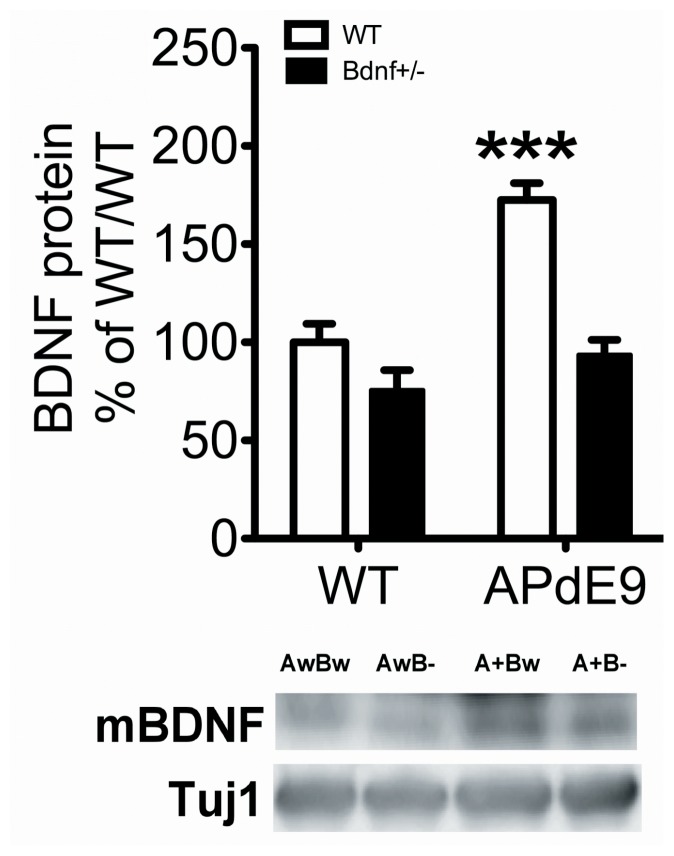
Mature BDNF protein levels are specifically increased in 13 month-old female *APdE9* mouse cortex. Western blot analysis of mature-BDNF protein levels in the cortex of wild-type mice and mice carrying *APdE9* and/or *Bdnf* ^+/−^ mutations. Two-Way ANOVA followed with Tukey-Kramer *post hoc* test was performed for statistical analysis. *p<0.05, ***p<0.001 compared to the respective wt, ###p<0.001 compared to wt/wt.

The ELISA method that we used for BDNF protein analysis preferentially detects mature-BDNF over pro-BDNF [33]. To confirm the ELISA experiments and to examine the potential changes of pro-BDNF levels in the brains of *APdE9* mice, we performed western blot analyses with an antibody that was confirmed to detect both mature and pro forms of BDNF in brain tissue samples (**Fig. S2**). In agreement with ELISA data, western blot analysis showed that the levels of mature BDNF were strongly increased in the temporal cortex of aged *APdE9* mice and *APdE9* x *Bdnf* ^+/−^ mice when compared to WT or *Bdnf* ^+/−^ mice, respectively (**Fig. 6**). The protein levels of pro-BDNF were extremely low or undetectable in all of the samples obtained from WT or mutant mice carrying *APdE9* and/or *Bdnf* mutations.

**Figure 7:**
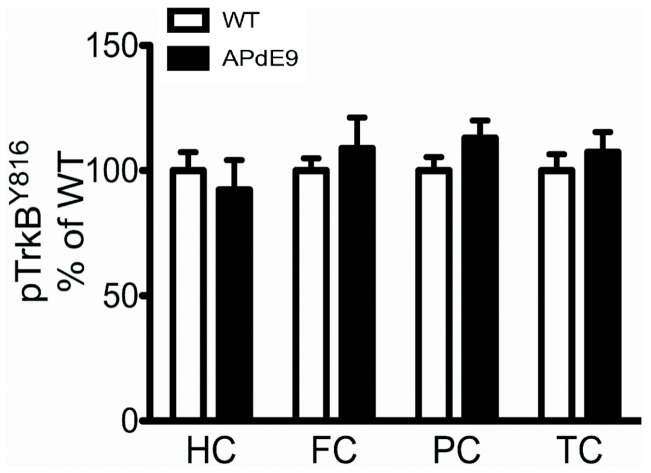
BDNF protein load is not associated with TrkB receptor activity in *APdE9* mouse cortex. TrkB receptor phosphorylation (Y816) levels in the hippocampus, frontal cortex, parietal cortex and temporal cortex of 12-month-old female *APdE9* mice.

Next, we tested if the relatively robust increase in the levels of mature BDNF protein in the cortex of aged *APdE9* mice leads to functional changes in TrkB receptor activity. However, the TrkB phosphorylation status remained unaltered in the hippocampal and cortical samples of 12-month-old *APdE9* mice (Y816, **Fig. 7**; Y705/6, **data not shown**).

### BDNF Protein Levels are Predominantly Increased around Amyloid Plaques in the Brains of Aged *APdE9* Mice

**Figure 8:**
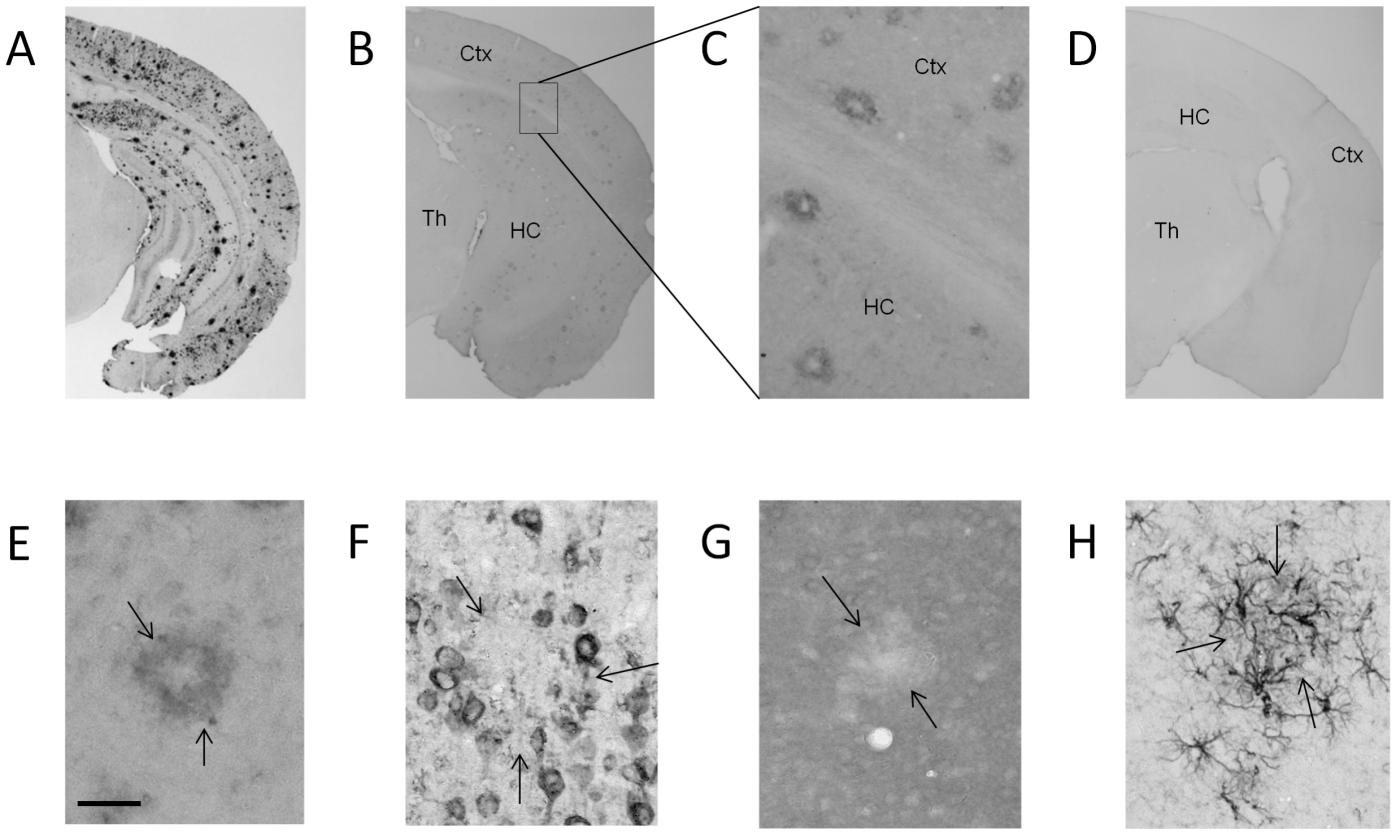
BDNF protein accumulates around amyloid plaques in the brains of ***APdE9*** mice. **A)** Amyloid-β selective antibody W02 detects a robust accumulation of amyloid-β in the brain of a 13-month-old *APdE9* mouse. **B)** BDNF antibody reveals significant anti-BDNF immunoreactivity in the cortex and hippocampus but not in the thalamus of aged *APdE9* mouse, thus matching the regional pattern of Amyloid-β accumulation. **C)** A magnified view of the framed area demonstrating doughnut-shaped anti-BDNF immunopositivity around amyloid plaques. **D)** The same BDNF antibody dilution that was used to identify BDNF accumulation in the Amyloid-β plaques in *APdE9* mouse brain did not show notable anti-BDNF immunoreactivity in a wild-type mouse brain. A close-up of a single amyloid plaque in a *APdE9* mouse stained for (**E**) anti-BDNF, (**F**) anti-NGF, (**G**) anti-CDNF and (**H**) GFAP to visualize activated astroglia. Only BDNF shows a donut-like staining pattern. Small arrows indicate plaque boundaries. (**E–H**) Scale bar = 50 µm. Th = thalamus, HC = hippocampus, Ctx = cerebral cortex.

Since a robust BDNF up-regulation in the cortex of aged *APdE9* mice was not associated with increased levels of *Bdnf* mRNA and enhanced TrkB signaling, it is unlikely that the increased BDNF protein resulted from increased BDNF synthesis. Rather BDNF might have accumulated into compartments where it could not be released to activate TrkB. Indeed, when we analyzed the immunohistochemical localization of BDNF in the brains of *APdE9* and WT mice, strong anti-BDNF immunoreactivity surrounded amyloid plaques in the hippocampus and cortex of *APdE9* mice in a “doughnut-like” fashion (**Fig. 8A–C**). In the absence of amyloid plaques such “doughnut-like” BDNF immunoreactivity could not be detected in WT mice (**Fig. 8D**). Furthermore, stainings for other neurotrophins (NGF and CDNF) or activated astroglia resulted in a very different staining pattern, ruling out the possibility that this is simply a results of nonspecific binding of antibodies to sticky amyloid plaques (**Fig. 8** **E–H**).

## Discussion

We addressed the role of BDNF in Alzheimer’s disease (AD) by investigating behavior and neurochemistry in double mutant mice generated by crossing BDNF-deficient (*Bdnf* ^+/−^) mice with an AD mouse model, *APdE9* mice. We have previously reported that inhibition of TrkB signaling aggravates memory impairment in *APdE9* mice, whereas increasing TrkB signaling ameliorates it [29]. In line with these findings, haploinsufficiency of *Bdnf* further exacerbated impaired spatial learning ability of *APdE9* mice. This deficit was most prominent in males. Similarly to *APdE9* mice, *Bdnf* ^+/−^ males displayed strong wall-hugging behavior (thigmotaxis) in the Morris swim task. However, in contrast to the task acquisition, *Bdnf* ^+/−^ gene deficiency in *APdE9* mice did not influence their memory for the platform location in the probe test.

One limitation of the present *Bdnf* ^+/−^ mouse model is the constitutive nature of the BDNF haploinsufficiency. Among other things, this led to significant obesity in *Bdnf* ^+/−^ mice as reported before [43]. Obesity was a likely confounding factor for escape latency in the Morris swim task (*via* decreased swimming speed), spontaneous exploratory activity and Rotarod performance among the male mice that showed the largest differences in body weight due to *Bdnf* gene deficiency. To circumvent the contribution of obesity to observed spatial learning deficit in *Bdnf* ^+/−^ mice, we took body weight as a cofactor in the statistical model when assessing escape latency. This approach abolished the *Bdnf* ^+/−^ associated difference in the swimming speed, but did not abate the difference in escape latency. In addition, we also analyzed the swim path length that is less susceptible to the confounding influence of swimming speed than the more common measure of escape latency for the task acquisition. This analysis separately for each sex showed a trend toward impairment in *Bdnf* ^+/−^ mice, while in the pooled analysis with both sexes resulted in a clearly significant *Bdnf* gene effect. These findings together with a highly significant increase in thigmotaxis among *Bdnf* ^+/−^ male mice strongly suggest that *Bdnf* ^+/−^ mice were impaired in the Morris swim task acquisition independent of obesity and decreased swimming speed associated with the *Bdnf* ^+/−^ genotype. However, for some reason the *Bdnf* gene deficiency no longer manifested as impairment during the probe task.

In a novel test cage, *Bdnf* ^+/−^ mutant mice did not differ from wild-type mice, whereas *APdE9* mice showed prominent hyperactivity, as shown before [29]. This hyperactivity was completely normalized by *Bdnf* gene deficiency. Again, this finding persisted even after the possible confounding effect of body weight was taken into account in the statistical model. Therefore, even if deficient BDNF signaling is predominantly associated with deleterious effects it can produce paradoxically beneficial effects in some specific neuronal systems and behaviors. This notion is further strengthened by our recent studies demonstrating that genetic inhibition of the TrkB signaling and *bdnf* gene deficiency counteracts the hyperactivity present in *APdE9* and *Fmr1* ^−/−^ mutant mice, respectively [29], [46]. Finally, male *Bdnf* ^+/−^ mice were no worse than other test groups in the Rotarod test despite prominent obesity, while female double mutant mice outperformed other genotypes.

BDNF protein and mRNA levels have been extensively studied in post-mortem brain tissues obtained from AD patients and in diverse animal models of AD [12]. Most of these data are favoring overall reduced BDNF synthesis in AD [17], [21], [22], [47], that is thought to arise from direct effects of amyloid-β (Aβ) on *Bdnf* synthesis [48]. In particular, high Aβ42/Aβ40 ratio and the formation of large SDS-stable Aβ oligomers in brain have been recently associated with more robust reduction of *Bdnf* synthesis in mouse models of AD [23]. Interestingly however, some experiments have reported paradoxical increase of BDNF (mRNA, protein) in the brains of amyloid plaque forming mice [44], [45]. The observed BDNF increase in these mice appears to be concentrated predominantly in reactive glial cells around amyloid-β plaques [44]. Indeed, one human report shows an increased BDNF immunoreactivity in senile plaques in post-mortem AD patients [49]. In line with these findings, we observed that BDNF protein levels were either down-regulated (female) or unaltered (male) in the cortex of *APdE9* mice before the onset of amyloid plaque formation (3 months). This was followed by an age-dependent increase in BDNF protein levels in several cortical areas in male and female *APdE9* mice. Our western blot analyses confirmed the ELISA experiments demonstrating increased levels of mature-BDNF protein in the neocortex of middle-aged *APdE9* mice, while the levels of pro-BDNF remained undetectable in the cortex in both *APdE9* and WT mice. We were unable to reproduce *Bdnf* mRNA induction in the cortical samples of *APdE9* mice as has previously shown by Burbach *et al* [44], but this may be due to the fact that we did not microdissect plaques specifically for the analysis as done in their study. In line with Szapacs *et al* [45] we saw an up-regulation of BDNF in the hippocampus of aged *APdE9* mice but this increase was significantly less than that seen in the cortex, consistent with more prominent amyloid load in cortical regions than in the hippocampus in this mouse model. Furthermore, in agreement with a previous report [44], significant BDNF immunoreactivity was predominantly seen around amyloid plaques in *APdE9* mice.

As hypothesized before, the observed up-regulation of BDNF synthesis around amyloid plaques of aged *APdE9* mice may be an attempt to provide neurotrophic support for degenerating neurons around the plaques [44], [49]. Alternatively, BDNF may get “trapped” in the amyloid plaques and escape degradation. Therefore, we examined whether this BDNF protein induction would lead to significant changes in TrkB receptor activation by analyzing the phosphorylation status of TrkB. However, TrkB phosphorylation at sites Y705/6 and Y816, both of which are readily activated by BDNF [6], remained unaltered in the cortical samples of aged *APdE9* mice that demonstrated almost 2-fold increase in mature-BDNF protein levels. Since BDNF through its TrkB receptor activates the phosphoinositol kinase 3– Akt kinase pathway, leading to a disinhibition of the most important tau kinase, glycogen synthase kinase 3β, we also assessed eventual changes in tau phosphorylation status in amyloid plaques using the AT8 antibody. This analysis did not show any significant change in tau phosphorylation, a finding also supported by recently published report by Castello *et al* [50]. These findings, collectively with the lack of concomitant increase in *Bdnf* mRNA levels, strongly suggest that a substantial part of BDNF around the amyloid plaques does not actively participate in signaling. This would also explain why an ostensible “normalization” of brain BDNF levels in *APdE9* x *Bdnf* ^+/−^ mice impaired their spatial learning ability in a way that is consistent with BDNF deficiency. In other words, *Bdnf* ^+/−^ genotype was associated with impaired spatial learning both in mice wild-type for the *APdE9* transgene with half of the total cortical BDNF levels and in *APdE9* mice with similar total cortical BDNF levels as double wild-type mice.

In conclusion, our results are compatible with the idea that reduced levels of functional BDNF through haploinsufficiency is deleterious for learning and memory. Our results also support some of the previous findings suggesting that BDNF may get stuck into amyloid plaques, which can explain the paradox that *APdE9* x *Bdnf* ^+/−^ mice with their BDNF levels within the “normal range” are similarly impaired in comparison with their *Bdnf* wild-type littermates as mice without the *APdE9* transgene. However, further studies are needed to confirm that BDNF is truly enriched in detergent resistant brain tissue fractions where the plaques are also observed. If such accumulation of BDNF would also happen in human AD brains, it would suggest that functional BDNF levels in the AD brains are even lower than the already compromised total BDNF protein or mRNA levels would indicate.

## Supporting Information

Figure S1 *Bdnf* gene deficiency aggravates memory impairment due to the *APdE9* transgene in the Morris swim task. Male and female mice are pooled. (**A)** Swim path length to the hidden platform; *** significant *APdE9* gene main effect (p<0.001, ANOVA-rm),^ §§^ *Bdnf* gene main effect (p = 0.008, ANOVA-rm), ^###^A+B- mice differ significantly from the AwBw control group (p<0.001, Dunnett’s post-hoc test), ^##^A+Bw mice differ significantly from the AwBw control group (p = 0.007, Dunnett’s post-hoc test). (**B)** Thigmotaxis calculated as percent time spent in the wall zone; *** significant *APdE9* gene main effect (p<0.001, ANOVA-rm), ^§§§^ significant *Bdnf* gene main effect (p<0.001, ANOVA-rm), ^###^ A+B- mice differ significantly from the AwBw control group (p<0.001, Dunnett’s post-hoc test), ^##^ A+Bw mice mice differ significantly from the AwBw control group (p = 0.006, Dunnett’s post-hoc test). Abbreviations for genotypes: AwBw = *wt x wt*; AwB- = *wt x Bdnf^+/−^*; A+Bw = *APdE9 x wt*; A+B- = *APdE9 x Bdnf^+/−^*.
(TIF)

Figure S2 Validation of BDNF protein analyses. (**A**) BDNF antibody (sc-546) readily detects endogenous mature-BDNF (straight line) and pro-BDNF (dash line) in the hippocampal tissue of cleavage-resistant BDNF knock-in mouse (ki) whereas no pro-BDNF is detected in wild-type (wt) mouse samples. Human recombinant mature-BDNF and pro-BDNF are loaded as controls in right. Asterics mark for unspecific bands recognized by anti-BDNF. (**B**) BDNF ELISA readily detects BDNF protein in hippocampal tissues of wild-type (WT) mice whereas no signal over background is observed in samples obtained from conditional BDNF knock-out (KO) mice. (**C**) Increased levels of mature BDNF protein are detected in hippocampal tissues obtained from mice chronically treated antidepressant fluoxetine (0.08 mg/ml in drinking water for 21 days, n = 8/group; for details see Ref. 35) with employed western and ELISA methods. A sample from conditional BDNF knock-out (KO) mice was run as control for western blot analyses to confirm the specific band corresponding to mature BDNF. Note that anti-BDNF recognizes an intense, but unspecific, band around the level of pro-BDNF. Human recombinant mature-BDNF and pro-BDNF are loaded as controls in right. Asterics mark for unspecific bands recognized by anti-BDNF. A t-test was performed for the data shown in panel (C); *p<0.05.
(TIF)
