# Supplementary figures and images for: The Impact of Bdnf Gene Deficiency to the Memory Impairment and Brain Pathology of APPswe/PS1dE9 Mouse Model of Alzheimer’s Disease
Source: PLoS One. 2013 Jul 3;8(7):e68722. doi: 10.1371/journal.pone.0068722 (PMC3700921; doi:10.1371/journal.pone.0068722)

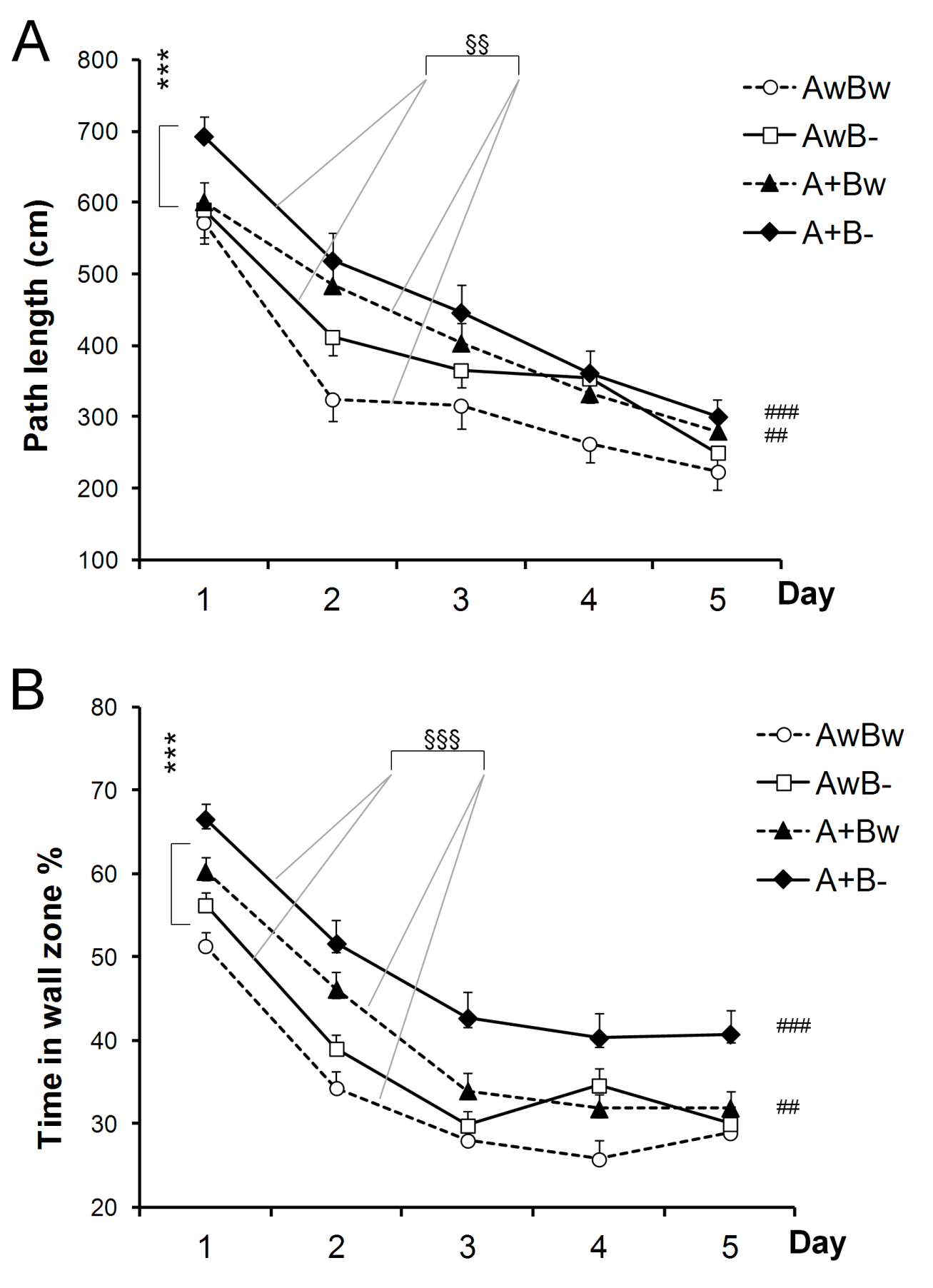

Supplement: Figure S1 — Bdnf gene deficiency aggravates memory impairment due to the APdE9 transgene in the Morris swim task. Male and female mice are pooled. (A) Swim path length to the hidden platform; *** significant APdE9 gene main effect (p<0.001, ANOVA-rm), §§ Bdnf gene main effect (p = 0.008, ANOVA-rm), ###A+B- mice differ significantly from the AwBw control group (p<0.001, Dunnett’s post-hoc test), ##A+Bw mice differ significantly from the AwBw control group (p = 0.007, Dunnett’s post-hoc test). (B) Thigmotaxis calculated as percent time spent in the wall zone; *** significant APdE9 gene main effect (p<0.001, ANOVA-rm), §§§ significant Bdnf gene main effect (p<0.001, ANOVA-rm), ### A+B- mice differ significantly from the AwBw control group (p<0.001, Dunnett’s post-hoc test), ## A+Bw mice mice differ significantly from the AwBw control group (p = 0.006, Dunnett’s post-hoc test). Abbreviations for genotypes: AwBw = wt x wt; AwB- = wt x Bdnf+/−; A+Bw = APdE9 x wt; A+B- = APdE9 x Bdnf+/−. (TIF) [file pone.0068722.s001.tif]

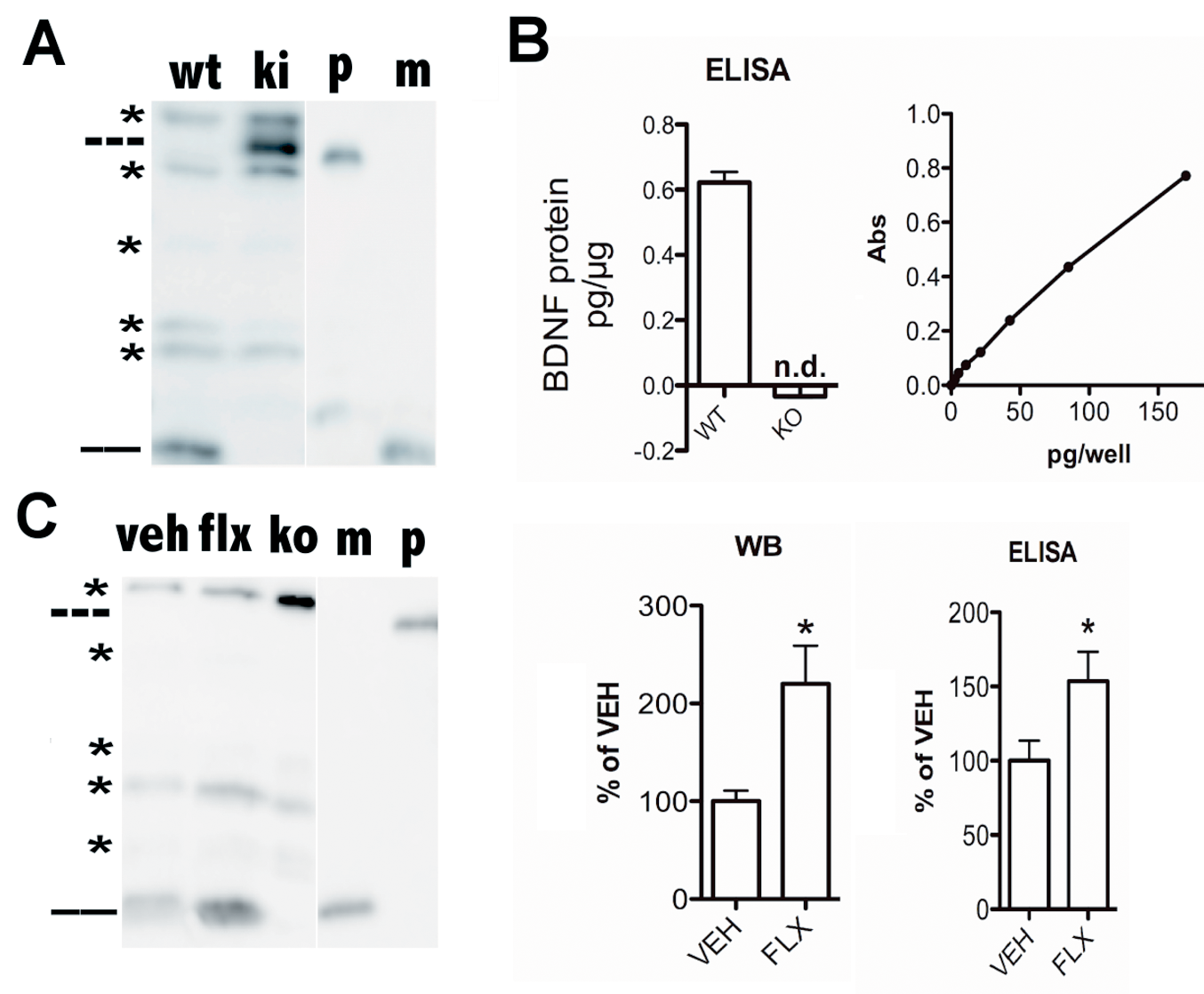

Supplement: Figure S2 — Validation of BDNF protein analyses. (A) BDNF antibody (sc-546) readily detects endogenous mature-BDNF (straight line) and pro-BDNF (dash line) in the hippocampal tissue of cleavage-resistant BDNF knock-in mouse (ki) whereas no pro-BDNF is detected in wild-type (wt) mouse samples. Human recombinant mature-BDNF and pro-BDNF are loaded as controls in right. Asterics mark for unspecific bands recognized by anti-BDNF. (B) BDNF ELISA readily detects BDNF protein in hippocampal tissues of wild-type (WT) mice whereas no signal over background is observed in samples obtained from conditional BDNF knock-out (KO) mice. (C) Increased levels of mature BDNF protein are detected in hippocampal tissues obtained from mice chronically treated antidepressant fluoxetine (0.08 mg/ml in drinking water for 21 days, n = 8/group; for details see Ref. 35) with employed western and ELISA methods. A sample from conditional BDNF knock-out (KO) mice was run as control for western blot analyses to confirm the specific band corresponding to mature BDNF. Note that anti-BDNF recognizes an intense, but unspecific, band around the level of pro-BDNF. Human recombinant mature-BDNF and pro-BDNF are loaded as controls in right. Asterics mark for unspecific bands recognized by anti-BDNF. A t-test was performed for the data shown in panel (C); *p<0.05. (TIF) [file pone.0068722.s002.tif]
